# Supplementary material for: Emergence of the East-Central-South-African genotype of Chikungunya virus in Brazil and the city of Rio de Janeiro may have occurred years before surveillance detection
Source: Sci Rep. 2019 Feb 26;9:2760. doi: 10.1038/s41598-019-39406-9 (PMC6391440; doi:10.1038/s41598-019-39406-9)
Supplement: Supplementary file 1 — Supplementary Material [file 41598_2019_39406_MOESM1_ESM.pdf]

SUPPLEMENTARY INFORMATION

Emergence of the East-Central-South-African genotype of Chikungunya virus in Brazil and the city of Rio de Janeiro may have occurred years before surveillance detection

Thiago Moreno L. Souza[1,5,11,*, #], Yasmine Rangel Vieira[1,#], Edson Delatorre[3,#], Giselle Barbosa-Lima[1], Raul Leal Faria Luiz[1], Alexandre Vizzoni[1], Komal Jain[2], Milene Mesquita[4], Nishit Bhuva[2], Jan F. Gogarten[2], James Ng[2], Riddhi Thakkar[2], Andrea Surrage Calheiros[5], Ana Paula Teixeira Monteiro[5], Patrícia T. Bozza[5], Fernando A. Bozza[1,6], Diogo A. Tschoeke[7], Luciana Leomil[7], Marcos Cesar Lima de Mendonça[8], Cintia Damasceno dos Santos Rodrigues[8], Maria C. Torres[8], Ana Maria Bispo de Filippis[8], Rita Maria Ribeiro Nogueira[8], Fabiano L. Thompson[7,9], Cristina Lemos[10], Betina Durovni[10], José Cerbino-Neto[1], Carlos M. Morel[11], W. Ian Lipkin[2,*, ¥,], Nischay Mishra[2, ¥,]

[#]- These authors contributed equally as first authors.

¥ - These authors contributed equally as last authors

*- Corresponding authors (see footnote)

Affiliations:

1 – Instituto Nacional de Infectologia (INI), Fundação Oswaldo Cruz (Fiocruz), Rio de Janeiro, RJ, Brazil.

2 – Center for Infection and Immunity, Mailman School of Public Health, Columbia University, New York, New York, USA.

3 – Laboratório de AIDS e imunologia Molecular, Instituto Oswaldo Cruz (IOC), Fiocruz, Rio de Janeiro, RJ, Brazil.

4 – Laboratório de Vírus Respiratório e do Sarampo, IOC, Fiocruz, Rio de Janeiro, RJ, Brazil.

5 – Laboratório de Imunofarmacologia, IOC, Fiocruz, Rio de Janeiro, RJ, Brazil.

6 – D'Or Institute for Research and Education (IDOR), Rio de Janeiro, RJ, Brazil.

7 – Instituto de Biologia, Universidade Federal do Rio de Janeiro (UFRJ), Rio de Janeiro, RJ, Brazil.

8 – Laboratório de Flavivírus, IOC, Fiocruz, Rio de Janeiro, RJ, Brazil.

9 – SAGE/COPPE, UFRJ, Rio de Janeiro, RJ, Brazil.

10 - Secretaria Municipal de Saúde, Rio de Janeiro, Brazil

11 – National Institute for Science and Technology on Innovation on Diseases of Neglected Populations (INCT/IDPN), Center for Technological Development in Health (CDTS), Fiocruz, Rio de Janeiro, RJ, Brazil.

* Corresponding authors:

Thiago Moreno L. Souza, Centro de Desenvolvimento Tecnológico em Saúde - CDTS, FIOCRUZ. Av. Brasil, n° 4365, Manguinhos - RJ. Cep: 21045-900. Rio de Janeiro, RJ, Brasil. Tel: (+ 55 21) 2562-1311; e-mail: tmoreno@cdts.fiocruz.br

W.Ian Lipkin, Center for Infection and Immunity, Mailman School of Public Health of Columbia University, 722 West 168th Street, New York, NY 10032, USA. Tel : (212) 342-9033 ; email : wil2001@cumc.columbia.edu

Supplemental Methodology

Downstream processing of the Full genome sequencing using VirCapSeq-VERT platform

Sequencing on the Illumina MiSeq platform (Illumina, San Diego, CA, USA) resulted in 30,393,722 (300 bp) paired end reads. The demultiplexed FastQ files were adapter trimmed using cutadapt program (v 1.8.3). Adaptor trimming was followed by generation of quality reports using FastQC software (v 0.11.5) which were used to determine filtering criteria based on average quality scores of the reads, presence of indeterminate nucleotides and homopolymeric reads. The reads were quality filtered and end-trimmed with PRINSEQ software (v 0.20.3). The filtered fastq files were mapped to the complete genome sequence of Chikungunya virus using Bowtie 2 mapper (v 2.2.9). The consensus genome sequences from mapping assemblies were obtained using SAM Tools (v 1.3.1) and Bcftools (v 1.3.1). The genome variation was analyzed using in-house perl scripts to count the frequency of nucleotides at each position with reference to the Chikungunya genome.

Read processing

The demultiplexed FastQ files were adapter trimmed using cutadapt program (v 1.8.3)[5]. Adaptor trimming was followed by generation of quality reports using FastQC software (v 0.11.5) which were used to determine filtering criteria based on average quality scores of the reads, presence of indeterminate nucleotides and homopolymeric reads[6]. Reads were quality filtered and end-trimmed with PRINSEQ software (v 0.20.3)[7]. The filtered fastq files were mapped to the complete genome sequence of CHIKV using Bowtie2 mapper (v 2.2.9)[8]. The consensus genome sequences from mapping assemblies were obtained using SAM Tools (v 1.3.1) and Bcftools (v 1.3.1)[9]. Genome variation was

analyzed using in-house perl scripts to count the frequency of nucleotides at each position with reference to the Chikungunya genome.

Unbiased-NGS

Additionally, two further CHIKV positive samples from 2015 were sequenced by unbiased shotgun sequencing. In brief, 0.3 mL plasma aliquot was extracted using QIAamp Viral RNA Mini Kit (Qiagen®) with RNase-free DNase (Qiagen®) treatment, omitting carrier RNA. Double-stranded cDNA libraries were constructed using a TruSeq Stranded Total RNA LT kit (Illumina®) with Ribo-zero treatment. The library size distribution was assessed using a 2100 Bioanalyzer (Agilent®) with a High Sensitivity DNA kit (Agilent®), and the quantification was performed using a 7500 Real-time PCR System (Applied Biosystems®) with a KAPA Library Quantification Kit (Kapa Biosystems). Paired-end sequencing (2 x 300 bp) was performed with a MiSeq Reagent kit v3 (Illumina®). The sequences obtained were preprocessed using the PRINSEQ software to remove reads smaller than 50 bp and sequences with scores of lower quality than a Phred quality score of 20. Paired-End reAd merger (PEAR) software was used to merge and extend the paired-end Illumina reads using the default parameter. The extended reads were analyzed against the Human Genome Database using the DeconSeq program, with an identity and coverage cutoff of 70%, to remove human RNA sequences. Non-human reads were analyzed against all GenBank viral genomes (65,052 sequences) using the BLAST software with a 1e-5 e-value cutoff. The sequences rendering a genome were assembled with SPAdes 3.7.1 software[10] followed by a second assembly with the CAP3 program using default parameters[11].

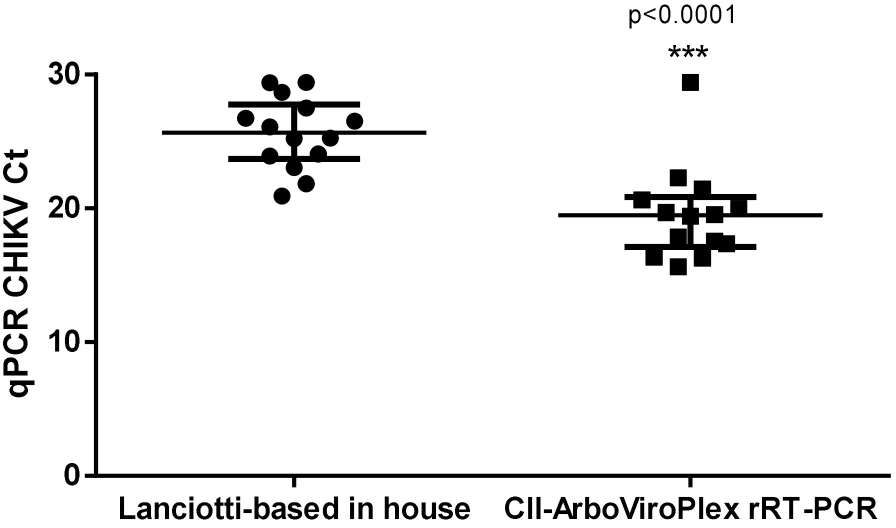

Figure S1. Comparison of diagnostic sensitivities among different acid nucleic extraction and RT-PCR methodologies. CHIKV cycle threshold (Ct) values obtained for 14 RNA samples collected between Feb 2016 - Feb 2017 processed by column-based extraction methods and qPCR *in house*[1–3] ranged from 20.92 to 29.42. Ct values obtained for the same samples after extraction methods based on magnetic beads and the new 5-ArboViroPlex qPCR[4] ranged from 16.3-29.41. Statistical analysis to compare sensitivities of CHIKV molecular diagnostics was performed in accordance with paired t test (95% confidence intervals). A p-value < 0.05 was considered statistically significant. CHIKV viral load values were significantly higher by using an automated purification method and the 5-ArboViroPlex qPCR ($p < 0.0001$).

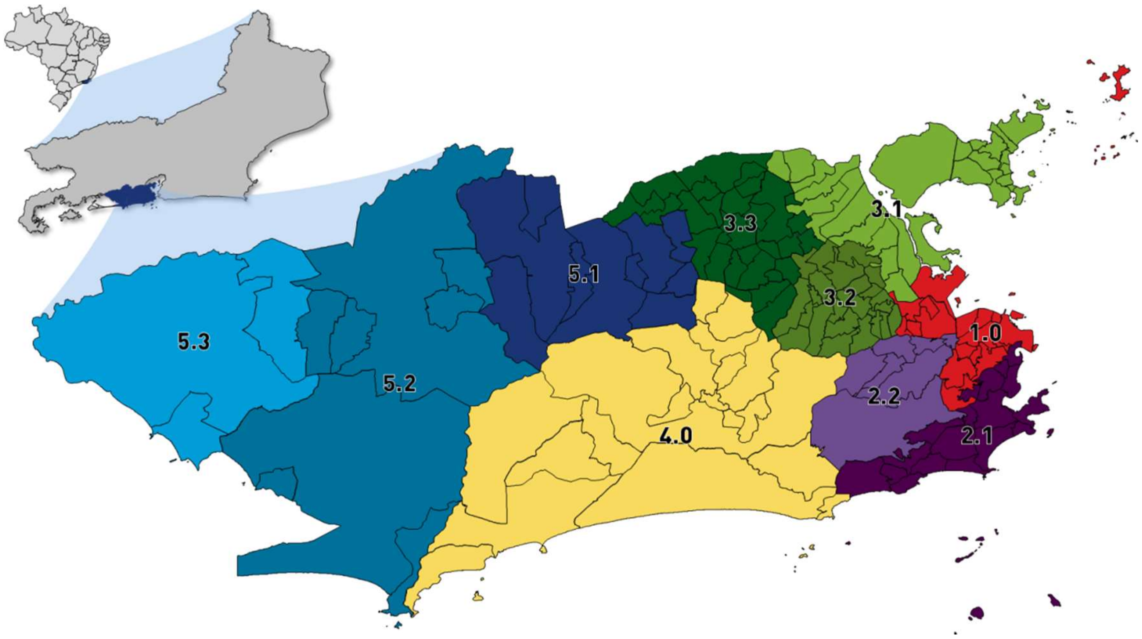

Figure S2. The City of Rio de Janeiro, Brazil and its planning areas (AP), distributed according to municipal administration.

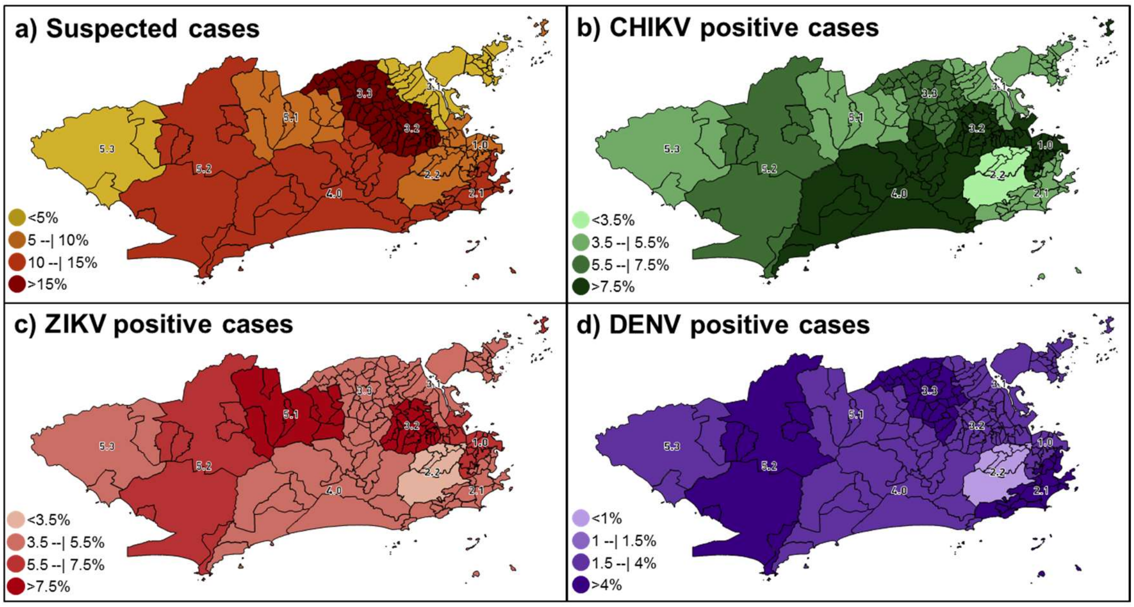

Figure S3. Geographic distribution of suspected and positive cases for arboviruses in the city of Rio de Janeiro, Brazil, from March 2016 to June 2017. Percentage of suspected (a) and positive cases for CHIKV (b), ZIKV (c) and DENV-4 (d) by PA. For guidance, the site where the patient was medically assisted was taken as reference. These percentages are not absolute indications that a certain PA is more affected by one virus than another. Because individuals may live in PA, work and seek for medical assistance in other regions. These data robustly show that cases of either DENV, ZIKV and CHIKV are widespread throughout the city.

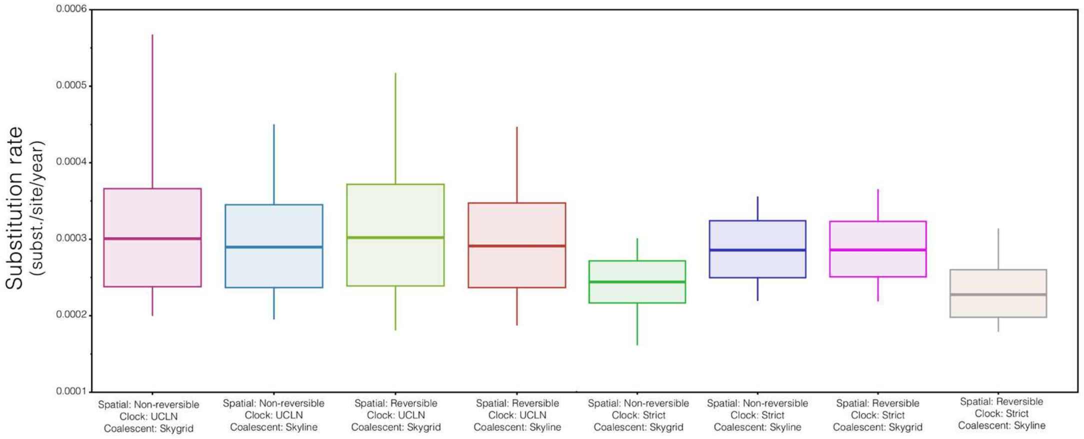

Figure S4. BEAST estimates of the CHIKV ECSA substitution rates employing different model combinations. The box plots represent the median substitution rates (substitutions/site/year) and the 95% Bayesian credible intervals of the posterior distributions estimated under each model combination as informed in the x-axis.

Table S1. *Description of published primer/probe sets used in qPCR.*

Virus Target	Reference	Primer/Probe	Genome Position	Sequence (5´- 3´)
ZIKV	Lanciotti *et al*, 2008[1]	Primer F	1086-1102	CCG CTG CCC AAC ACA AG
		Primer R	1162-1139	CCA CTA ACG TTC TTT TGC AGA CAT
		Probe	1107-1137	FAM/ AGC CTA CCT TGA CAA GCA GTC AGA CAC TCA A /BHQ1
CHIKV	Lanciotti *et al*, 2007[2]	Primer F	874-894	AAA GGG CAA ACT CAG CTT CAC
		Primer R	961-942	GCC TGG GCT CAT CGT TAT TC
		Probe	899-923	FAM/ CGC TGT GAT ACA GTG GTT TCG TGT G/BHQ1
DENV1	Santiago *et al*, 2013[3]	Primer F	8936-8955	CAA AAG GAA GTC GYG CAA TA
		Primer R	9023-9047	CTG AGT GAA TTC TCT CTG CTR AAC
		Probe	8961-8979	FAM/CAT GTG GYT GGG AGC RCG C/BHQ1
DENV2	Santiago *et al*, 2013[3]	Primer F	1426-1447	CAG GCT ATG GCA CYG TCA CGA T
		Primer R	1482-1504	CCA TYT GCA GCA RCA CCA TCT C
		Probe	1454-1480	VIC/CTC YCC RAG AAC GGG CCT CGA CTT CAA/BHQ1
DENV3	Santiago *et al*, 2013[3]	Primer F	701-720	GGA CTR GAC ACA CGC ACC CA
		Primer R	749-775	CAT GTC TCT ACC TTC TCG ACT TGY CT
		Probe	722-747	FAM/ACC TGG ATG TCG GCT GAA GGA GCY TG/BHQ1
DENV4	Santiago *et al*, 2013[3]	Primer F	884-904	TTG TCC TAA TGA TGC TRG TCG
		Primer R	953-973	TCC ACC YGA GAC TCC TTC CA
		Probe	939-965	FAM/TYC CTA CYC CTA CGC ATC GCA TTC CG/BHQ1

**Bibliographical references are indicated at the end of this file*

Table S2 - Quality control of genome assembly

Sample Name	GenBank ID	Collection date	Collection site*	Lanciotti-based in house Cts	CII Arboviro-Plex Cts	Sequence technology	Number rawfastq reads	Number input reads	Number mapped reads	Mapped reads (%)	Genome length	CHIKV used for mapping reference	Refseq Length	Number Mapped Pos Ref (Length Genome Recovered)	Genome Recovered (%)	Avg. depth/bp	Avg. unmapped length	Longest Unmapped Start	Longest Unmapped End
BRZ-10_S8_L001_R1_001	MG649972	May 04, 2016	PA 1.0	26.08	16.35	ViralCapSeq-VERT	2,71E+06	2,65E+06	7,86E+05	29.71	1,17E+04	NC_004162.2	1,18E+04	1,18E+04	99.72	9299	33	1,18E+04	1,18E+04
BRZ-12_S9_L001_R1_001	MG649982	Mar 19, 2017	PA 5.2	26.50	19.54	ViralCapSeq-VERT	2,27E+06	2,19E+06	1,01E+06	46.26	1,28E+04	NC_004162.2	1,18E+04	1,18E+04	100	12082	NA	NA	NA
BRZ-14_S10_L001_R1_001	MG649978	Mar 14, 2017	PA 5.2	26.74	19.68	ViralCapSeq-VERT	2,51E+06	2,41E+06	2,54E+05	10.53	1,17E+04	NC_004162.2	1,18E+04	1,18E+04	99.81	3104	22	1,18E+04	1,18E+04
BRZ-18_S11_L001_R1_001	MG649971	Aug 21, 2016	PA 3.3	27.48	21.44	ViralCapSeq-VERT	1,39E+06	1,31E+06	1,69E+04	1.28	1,17E+04	NC_004162.2	1,18E+04	1,18E+04	99.66	202	40	1,18E+04	1,18E+04
BRZ-1_S1_L001_R1_001	MG649977	July 27, 2016	PA 5.2	20.92	17.55	ViralCapSeq-VERT	2,27E+06	2,20E+06	2,54E+05	11.51	1,17E+04	NC_004162.2	1,18E+04	1,18E+04	99.79	3007	24	1,18E+04	1,18E+04
BRZ-20_S12_L001_R1_001	MG649976	Mar 28, 2016	PA 1.0	28.66	19.98	ViralCapSeq-VERT	2,03E+06	1,96E+06	7,80E+04	3.99	1,17E+04	NC_004162.2	1,18E+04	1,18E+04	99.75	906	29	1,18E+04	1,18E+04
BRZ-2_S2_L001_R1_001	MG649981	Jul 27, 2016	PA 3.2	21.84	17.85	ViralCapSeq-VERT	1,50E+06	1,44E+06	8,19E+04	5.69	1,17E+04	NC_004162.2	1,18E+04	1,18E+04	99.96	982	4	1,18E+04	1,18E+04
BRZ-37_S13_L001_R1_001	MG649973	Dec 12, 2016	PA 4.0	29.40	22.30	ViralCapSeq-VERT	1,88E+06	1,82E+06	1,21E+05	6.63	1,17E+04	NC_004162.2	1,18E+04	1,18E+04	99.72	1363	32	1,18E+04	1,18E+04
BRZ-38_S14_L001_R1_001	MG649970	Mar 29, 2016	PA 1.0	29.42	20.63	ViralCapSeq-VERT	1,21E+06	1,17E+06	4,01E+04	3.44	1,17E+04	NC_004162.2	1,18E+04	1,18E+04	99.56	477	51	1,18E+04	1,18E+04
BRZ-3_S3_L001_R1_001	MG649974	Apr 07, 2016	PA 1.0	23.04	15.63	ViralCapSeq-VERT	3,66E+06	3,59E+06	4,00E+05	11.13	1,17E+04	NC_004162.2	1,18E+04	1,18E+04	99.73	4570	31	1,18E+04	1,18E+04
BRZ-4_S4_L001_R1_001	MG649975	Jul 24, 2016	PA 3.2	23.91	17.36	ViralCapSeq-VERT	1,72E+06	1,67E+06	1,52E+05	9.09	1,17E+04	NC_004162.2	1,18E+04	1,18E+04	99.74	1806	30	1,18E+04	1,18E+04
BRZ-6_S5_L001_R1_001	MG649983	Mar 28, 2016	PA 1.0	24.07	19.42	ViralCapSeq-VERT	2,22E+06	2,16E+06	6,74E+04	3.12	1,28E+04	NC_004162.2	1,18E+04	1,18E+04	100	751	NA	NA	NA
BRZ-7_S6_L001_R1_001	MG649980	Apr 26, 2016	PA 1.0	25.20	16.30	ViralCapSeq-VERT	2,35E+06	2,28E+06	8,73E+05	38.29	1,17E+04	NC_004162.2	1,18E+04	1,18E+04	99.94	10198	7	1,18E+04	1,18E+04
BRZ-8_S7_L001_R1_001	MG649979	Mar 03, 2017	PA 1.0	25.24	29.41	ViralCapSeq-VERT	2,66E+06	2,58E+06	3,29E+05	12.74	1,17E+04	NC_004162.2	1,18E+04	1,18E+04	99.89	3880	13	1,18E+04	1,18E+04
CHK_5_Brazil-RJ_2015	MG649984	2015	ND	ND	ND	Unbiased sequence	2,15E+06	1,98E+06	1,93E+05	9.73	1,12E+04	No reference	No reference	1,20E+04	100	3790	NA	NA	NA
CHK_7_Brazil-RJ_2015	MG649985	2015	ND	ND	ND	Unbiased sequence	8,43E+05	7,64E+05	5,18E+02	0.07	1,12E+04	No reference	No reference	1,12E+04	95.02	19.72	NA	NA	NA

*Reference to Figure S3

Legend:

ND - Not determined (donated by Arbovirus Reference Laboratory and derived from their surveillance)

NA - Not applicable

Table S3. Chikungunya virus complete-genome sequences used for phylogenetic analyses.

Acession Number	Analysis	Country	Host	Isolation source	Colection date	Passage history
KR559493	ML	American Samoa			2014	
HM045823	ML	Angola			1962	
KY435455	ML	Anguilla	Homo sapiens	cell culture supernatant (vero)	12/nov/14	Passage in cell culture
KY435483	ML	Anguilla	Homo sapiens	cell culture supernatant (vero)	12-Feb-2014	Passage in cell culture
KY435479	ML	Antigua - Barbuda	Homo sapiens	cell culture supernatant (vero)	28-Apr-2014	Passage in cell culture
KY435470	ML	Bahamas	Homo sapiens	cell culture supernatant (vero)	08/jul/14	Passage in cell culture
FJ807898	ML	Bangladesh	Homo sapiens	infected patient	2008	
KU365370	ML	Bangladesh	Homo sapiens		nov/11	
KU365371	ML	Bangladesh	Homo sapiens		nov/11	
KY435464	ML	Barbados	Homo sapiens	cell culture supernatant (vero)	15-Aug-2014	Passage in cell culture
KY435466	ML	Barbados	Homo sapiens	cell culture supernatant (vero)	06-Aug-2014	Passage in cell culture
KP164568	ML	Brazil	Homo sapiens		26-Aug-2014	
KP164570	ML	Brazil	Homo sapiens		03-Sep-2014	
KP164571	ML	Brazil	Homo sapiens		03/jul/14	
KP164572	ML	Brazil	Homo sapiens		21-Aug-2014	

KT581 023	ML	Brazil	Homo sapiens		2014	
KU355 832	ML	Brazil	Homo sapiens		2015	
KU940 225	ML	Brazil	Homo sapiens		15/jul /15	
KU940 226	ML	Brazil	Homo sapiens		01- Aug- 2015	
KX228 391	ML	Brazil	Homo sapiens		03/ma r/16	
KY055 011	ML	Brazil	Aedes aegypti		20- Feb- 2016	
KY704 933	ML /Ba yes	Brazil	Homo sapiens		30/ma r/16	
KY704 934	ML /Ba yes	Brazil	Homo sapiens		30/ma r/16	
KY704 935	ML /Ba yes	Brazil	Homo sapiens		01- Apr- 2016	
KY704 936	ML /Ba yes	Brazil	Homo sapiens		01- Apr- 2016	
KY704 937	ML /Ba yes	Brazil	Homo sapiens		15- Apr- 2016	
KY704 938	ML /Ba yes	Brazil	Homo sapiens		19- Apr- 2016	
KY704 939	ML /Ba yes	Brazil	Homo sapiens		17- Apr- 2016	
KY704 940	ML /Ba yes	Brazil	Homo sapiens		14- Apr- 2016	
KY704 941	ML /Ba yes	Brazil	Homo sapiens		14- Apr- 2016	
KY704 942	ML /Ba yes	Brazil	Homo sapiens		16- Apr- 2016	

KY704 943	ML /Ba yes	Brazil	Homo sapiens		19-Apr-2016	
KY704 944	ML /Ba yes	Brazil	Homo sapiens		19-Apr-2016	
KY704 945	ML /Ba yes	Brazil	Homo sapiens		14-Apr-2016	
KY704 946	ML /Ba yes	Brazil	Homo sapiens		17-Apr-2016	
KY704 947	ML /Ba yes	Brazil	Homo sapiens		15-Apr-2016	
KY704 948	ML /Ba yes	Brazil	Homo sapiens		19-Apr-2016	
KY704 949	ML /Ba yes	Brazil	Homo sapiens		07-Apr-2016	
KY704 950	ML /Ba yes	Brazil	Homo sapiens		07-Apr-2016	
KY704 951	ML /Ba yes	Brazil	Homo sapiens		13-Apr-2016	
KY704 952	ML /Ba yes	Brazil	Homo sapiens		07-Apr-2016	
KY704 953	ML /Ba yes	Brazil	Homo sapiens		09-Apr-2016	
KY704 954	ML /Ba yes	Brazil	Homo sapiens		20/jun /16	
KY704 955	ML /Ba yes	Brazil	Homo sapiens		17/jun /16	
KP164 567	ML	Brazil-AP	Homo sapiens		28-Aug-2014	
KP164 569	ML	Brazil-BA	Homo sapiens		28-Aug-2014	

KY124328	ML	Brazil-RJ	Homo sapiens	patient with febrile illness	16/mar/16	
KY124329	ML	Brazil-RJ	Homo sapiens	patient with febrile illness	16/mar/16	
KJ451624	ML	British Virgin	Homo sapiens		jan/14	
KY435486	ML	British Virgin	Homo sapiens	cell culture supernatant (vero)	23/jan/14	Passage in cell culture
JQ861253	ML	Cambodia	Homo sapiens		16-Aug-2011	
JQ861254	ML	Cambodia	Homo sapiens		16-Aug-2011	
JQ861255	ML	Cambodia	Homo sapiens		16-Aug-2011	
JQ861256	ML	Cambodia	Homo sapiens		16-Aug-2011	
JQ861257	ML	Cambodia	Homo sapiens		16-Aug-2011	
JQ861258	ML	Cambodia	Homo sapiens		16-Aug-2011	
JQ861259	ML	Cambodia	Homo sapiens		26-May-2011	
JQ861260	ML	Cambodia	Homo sapiens		28-May-2011	
KX262996	ML/Bayes	Cameroon	Homo sapiens		2006	passage history: Vero 3; genotype: East-Central-South-African
HM045784	ML/Bayes	CAR	Anopheles (Ceilia) funestus		19/nov/84	
HM045822	ML/Bayes	CAR	Homo sapiens		Oct-1978	
KY038946	ML/Bayes	CAR	Aedes opok		jun/75	viral sample derived from a lyophilized vial

KY038947	ML/Bayes	CAR	Homo sapiens		Dec-1983	viral sample derived from a lyophilized vial; CRORAcollection
KY435459	ML	Cayman-Islands	Homo sapiens	cell culture supernatant (vero)	17-Sep-2014	Passage in cell culture
KY435460	ML	Cayman-Islands	Homo sapiens	cell culture supernatant (vero)	06/jul/14	Passage in cell culture
GU199350	ML	China	Homo sapiens	serum	2008	
GU199351	ML	China	Homo sapiens	serum	2008	passaged once in Vero cells
GU199352	ML	China	Homo sapiens	serum	2008	
GU199353	ML	China	Homo sapiens	serum	2008	
HQ846356	ML	China	Homo sapiens	serum	Oct-2010	
HQ846357	ML	China	Homo sapiens	serum	Oct-2010	
HQ846358	ML	China	Homo sapiens	serum	Oct-2010	
HQ846359	ML	China	Homo sapiens	serum	Oct-2010	
JQ065885	ML	China	Homo sapiens		Oct-2010	
JQ065886	ML	China	Homo sapiens		Oct-2010	
JQ065887	ML	China	Homo sapiens		Oct-2010	
JQ065888	ML	China	Homo sapiens		Oct-2010	
JQ065889	ML	China	Homo sapiens		Oct-2010	
JQ065890	ML	China	Homo sapiens		Oct-2010	
JQ065891	ML	China	Homo sapiens		Oct-2010	
JQ065892	ML	China	Homo sapiens		Oct-2010	
JX088705	ML	China	Homo sapiens		2010	

KC488650	ML	China	Homo sapiens	infected patient	2012	
KF318729	ML	China	Homo sapiens		06/jul/12	
KR559491	ML	Colombia			Aug-2014	
KU365372	ML	Colombia	Homo sapiens		18-Dec-2014	
KU365373	ML	Colombia	Homo sapiens		Dec-2014	
KX496989	ML	Colombia	Homo sapiens	blood	09-Feb-2016	
KF283986	ML	Comoros	Homo sapiens		2005	
KF283987	ML	Comoros	Homo sapiens		2005	
KP702297	ML	Comoros	Homo sapiens		2005	
KP003813	ML/Bayes	Congo	Homo sapiens		2011	
HM045818	ML	Cote-dIvoire	Aedes luteocephalus		Sep-1981	
HM045820	ML	Cote-dIvoire	Aedes africanus		Dec-1993	
KY435484	ML	Dominica	Homo sapiens	cell culture supernatant (vero)	30/jan/14	Passage in cell culture
KY435485	ML	Dominica	Homo sapiens	cell culture supernatant (vero)	28/jan/14	Passage in cell culture
KR559477	ML	Dominican-Republic			jul/14	
KR559479	ML	Dominican-Republic			Apr-2014	
KR559498	ML	Dominican-Republic			mar/14	

KY272 961	ML	Dominic an-Republi c	Homo sapiens	sera	2014	
KY272 962	ML	Dominic an-Republi c	Homo sapiens	sera	2014	
KY272 963	ML	Dominic an-Republi c	Homo sapiens	sera	2014	
KY272 964	ML	Dominic an-Republi c	Homo sapiens	sera	2014	
KY272 965	ML	Dominic an-Republi c	Homo sapiens	sera	2014	
KY272 966	ML	Dominic an-Republi c	Homo sapiens	sera	2014	
KY272 967	ML	Dominic an-Republi c	Homo sapiens	sera	2014	
KY272 968	ML	Dominic an-Republi c	Homo sapiens	sera	2014	
KY272 969	ML	Dominic an-Republi c	Homo sapiens	sera	2014	
KY272 970	ML	Dominic an-Republi c	Homo sapiens	sera	2014	
HM045 809	ML /Ba yes	DRC	Homo sapiens		1960	
KR559 471	ML	El-Salvado r			Oct-2014	

KR559472	ML	El-Salvador			jun/14	
KR559475	ML	El-Salvador			Sep-2014	
KR559484	ML	El-Salvador			nov/14	
KP003807	ML	France	Homo sapiens		2006	
KR559473	ML	French Polynesia			Feb-2015	
KX262994	ML	French-Guiana	Homo sapiens		21/jan/14	passage history: C636#2, Vero 1
KP003812	ML/Bayes	Gabon	Homo sapiens		2007	
KY435469	ML	Grenada	Homo sapiens	cell culture supernatant (vero)	30/jul/14	Passage in cell culture
KY435472	ML	Grenada	Homo sapiens	cell culture supernatant (vero)	16/jun/14	Passage in cell culture
KX262992	ML	Guadeloupe	Homo sapiens		05/jan/14	passage history: C636#2, Vero 1
LN898094	ML	Guadeloupe	Homo sapiens		jan/14	
LN898097	ML	Guadeloupe	Homo sapiens		jan/14	
LN898098	ML	Guadeloupe	Homo sapiens		jan/14	
LN898099	ML	Guadeloupe	Homo sapiens		jan/14	
LN898102	ML	Guadeloupe	Homo sapiens		jan/14	
LN898103	ML	Guadeloupe	Homo sapiens		jan/14	
LN898110	ML	Guadeloupe	Homo sapiens		jan/14	
LN898111	ML	Guadeloupe	Homo sapiens		jan/14	
KR559481	ML	Guatemala			Sep-2014	

KR559 490	ML	Guyana			Aug-2014	
KR559 496	ML	Guyana			jul/14	
KY435 458	ML	Guyana	Homo sapiens	cell culture supernatant (vero)	03/nov/14	Passage in cell culture
KY435 477	ML	Guyana	Homo sapiens	cell culture supernatant (vero)	31-May-2014	Passage in cell culture
KY435 478	ML	Guyana	Homo sapiens	cell culture supernatant (vero)	17-May-2014	Passage in cell culture
KR559 476	ML	Haiti			jul/14	
KR559 478	ML	Haiti			May-2014	
KX702 401	ML	Haiti	Homo sapiens	plasma	02/jun/14	
KX702 402	ML	Haiti	Homo sapiens	plasma	09/jun/14	
KY415 978	ML	Haiti	Homo sapiens	plasma	29-May-2014	
KY415 979	ML	Haiti	Homo sapiens	plasma	29-May-2014	
KY415 980	ML	Haiti	Homo sapiens	plasma	05/jun/14	
KY415 981	ML	Haiti	Homo sapiens	plasma	10/jun/14	
KY415 982	ML	Haiti	Homo sapiens	plasma	11/jun/14	
KY415 983	ML	Haiti	Homo sapiens	plasma	02/jun/14	
KY415 984	ML	Haiti	Homo sapiens	plasma	24/jun/14	
KY415 985	ML	Haiti	Homo sapiens	plasma	13-Aug-2014	
KY435 480	ML	Haiti	Homo sapiens	cell culture supernatant (vero)	27-Apr-2014	Passage in cell culture

KR559487	ML	Honduras			jul/14	
KR559488	ML	Honduras			Sep-2014	
EF210157	ML	India	Homo sapiens		2006	
EU372006	ML	India	Homo sapiens		11/jun/07	
EU564335	ML	India	Homo sapiens	supernatant of infected Vero B4 cells	31-Oct-2006	passaged in Vero B4 cells
FJ000062	ML	India	Homo sapiens	CSF	Sep-2006	
FJ000063	ML	India	Homo sapiens	CSF	Oct-2006	
FJ000064	ML	India	Homo sapiens	CSF	Sep-2006	
FJ000065	ML	India	Homo sapiens	CSF	Sep-2006	
FJ000066	ML	India	Homo sapiens	serum	Sep-2006	
FJ000067	ML	India	Homo sapiens	CSF	Aug-2006	
FJ000068	ML	India	Homo sapiens	CSF	Aug-2006	
FJ000069	ML	India	Homo sapiens	serum	jun/07	
GQ428210	ML	India	Homo sapiens	serum	07-Oct-2006	
GQ428211	ML	India	Homo sapiens	serum	07-Oct-2006	
GQ428212	ML	India	Homo sapiens	serum	12/jul/07	
GQ428213	ML	India	Homo sapiens	serum	13/jul/07	
GQ428214	ML	India	Homo sapiens	serum	29/jun/08	
GQ428215	ML	India	Homo sapiens	serum	29-May-2008	

HM045 788	ML	India	Homo sapiens		1973	
HM045 803	ML	India	Homo sapiens		06/no v/63	
HM045 806	ML	India			1986	
HM045 813	ML	India	Homo sapiens		06/no v/63	
JF2740 82	ML	India	Homo sapiens	serum	27-Sep-2006	
JN5588 34	ML	India	Homo sapiens		2009	
JN5588 35	ML	India	Homo sapiens		2008	
JN5588 36	ML	India	Homo sapiens		2009	
KJ6795 77	ML	India	Homo sapiens	serum of a patient visiting Calcutta School of Tropical Medicine	12-Sep-2011	
KJ6795 78	ML	India	Homo sapiens	serum of a patient visiting Calcutta School of Tropical Medicine	21-Dec-2011	
KJ7968 44	ML	India		serum	04-Aug-2009	
KJ7968 45	ML	India		serum	04-Aug-2009	
KJ7968 46	ML	India		serum	18-Aug-2009	
KT336 777	ML	India		human serum	08-Apr-2009	
KT336 778	ML	India		human serum	16/jul /10	
KT336 779	ML	India		human serum	03-Aug-2012	
KT336 780	ML	India		human serum	03-Aug-2012	

KT336781	ML	India		human serum	06/mar/13	
KT336782	ML	India		human serum	27-May-2013	
KX619424	ML	India	Homo sapiens	serum	22-Sep-2015	
KX619425	ML	India	Homo sapiens	serum	11-Sep-2015	
KX619426	ML	India	Homo sapiens	serum	03-Dec-2015	
KY057363	ML	India	Homo sapiens		28-Aug-2016	
KY751908	ML	India	male patient		2016	
FJ807897	ML	Indonesia	Homo sapiens	infected patient	2007	
HM045791	ML	Indonesia	Homo sapiens		1983	
HM045797	ML	Indonesia	Homo sapiens		1985	
KC862329	ML	Indonesia	Homo sapiens	serum	2010	
KM673291	ML	Indonesia	Homo sapiens	serum	jan/13	
EU244823	ML	Italy			2007	
KP003810	ML	Italy	Homo sapiens		2007	
KP003811	ML	Italy	Homo sapiens		2007	
KX262989	ML	Italy	Homo sapiens		2007	passage history: P1, Vero 1; genotype: East-Central-South-African
KX262993	ML	Italy	Homo sapiens		2007	passage history: P1(3308), Vero 1
KR559489	ML	Jamaica			Oct-2014	

KY435 461	ML	Jamaica	Homo sapiens	cell culture supernatant (vero)	24-Aug-2014	Passage in cell culture
KY435 462	ML	Jamaica	Homo sapiens	cell culture supernatant (vero)	25-Aug-2014	Passage in cell culture
KY435 468	ML	Jamaica	Homo sapiens	cell culture supernatant (vero)	06-Aug-2014	Passage in cell culture
FR717 336	ML	La-Reunion	Homo sapiens	host serum	26-Dec-2005	
FR717 337	ML	La-Reunion	Homo sapiens	host cornea	26-Dec-2005	
KP003 808	ML	Madaga scar	Homo sapiens		2006	
EU703 759	ML	Malaysi a	Homo sapiens	infected patient	2006	passaged twice in C636 cells
EU703 760	ML	Malaysi a	Homo sapiens	infected patient	2006	passaged twice in C636 cells
EU703 761	ML	Malaysi a	Homo sapiens	infected patient	2006	passaged twice in C636 cells
EU703 762	ML	Malaysi a	Homo sapiens	infected patient	2006	passaged twice in C636 cells
FJ8078 99	ML	Malaysi a	Homo sapiens	infected patient	2008	
FN295 483	ML	Malaysi a	Homo sapiens	host serum	mar/0 6	
FN295 484	ML	Malaysi a	Homo sapiens	host serum	mar/0 6	
FN295 485	ML	Malaysi a	Homo sapiens	host serum	2008	
FN295 487	ML	Malaysi a	Homo sapiens	host serum	2008	
FR687 340	ML	Malaysi a	Homo sapiens	human serum	21-Aug-2008	
FR687 341	ML	Malaysi a	Homo sapiens	human serum	05/no v/08	
FR687 342	ML	Malaysi a	Homo sapiens	human serum	10/no v/08	

FR687 343	ML	Malaysi a	Homo sapiens	human serum	12-Dec-2008	
FR687 344	ML	Malaysi a	Homo sapiens	human serum	12/jan /09	
FR687 345	ML	Malaysi a	Homo sapiens	human serum	05-Feb-2009	
FR687 346	ML	Malaysi a	Homo sapiens	human serum	16-Feb-2009	
FR687 347	ML	Malaysi a	Homo sapiens	human serum	12-Feb-2009	
FR687 348	ML	Malaysi a	Homo sapiens	human serum	03-Apr-2009	
KM923 917	ML	Malaysi a	Macaca fascicularis		09/ma r/07	
KM923 918	ML	Malaysi a	Macaca fascicularis		09/ma r/07	
KM923 919	ML	Malaysi a	Macaca fascicularis		09/ma r/07	
KM923 920	ML	Malaysi a	Macaca fascicularis		09/ma r/07	
KX168 429	ML	Malaysi a	Homo sapiens	serum	2009	
KX262 997	ML	Malaysi a	Homo sapiens		2009	passage history: C636#1
LN898 093	ML	Martiniq ue	Homo sapiens		Dec-2013	
LN898 095	ML	Martiniq ue	Homo sapiens		jan/14	
LN898 096	ML	Martiniq ue	Homo sapiens		jan/14	
LN898 100	ML	Martiniq ue	Homo sapiens		jan/14	
LN898 101	ML	Martiniq ue	Homo sapiens		jan/14	
LN898 104	ML	Martiniq ue	Homo sapiens		jan/14	
LN898 105	ML	Martiniq ue	Homo sapiens		jan/14	

LN898106	ML	Martinique	Homo sapiens		jan/14	
LN898107	ML	Martinique	Homo sapiens		jan/14	
LN898108	ML	Martinique	Homo sapiens		jan/14	
LN898109	ML	Martinique	Homo sapiens		jan/14	
LN898112	ML	Martinique	Homo sapiens		jan/14	
EU564334	ML	Mauritius	Homo sapiens	supernatant of infected Vero B4 cells	14-Feb-2006	passaged in Vero B4 cells
FJ959103	ML	Mauritius	Homo sapiens		2006	
KP003809	ML	Mayotte	Homo sapiens		2006	
KP851709	ML	Mexico	Homo sapiens; female	serum	15-Oct-2014	
KP851710	ML	Mexico	Homo sapiens; female	cell culture	30-May-2014	Passage in cell culture
KT327163	ML	Mexico	Homo sapiens	serum	2014	
KT327164	ML	Mexico	Homo sapiens	serum	2014	
KT327165	ML	Mexico	Homo sapiens	serum	2014	
KT327166	ML	Mexico	Homo sapiens	serum	2014	
KT327167	ML	Mexico	Homo sapiens	serum	2014	
KU365366	ML	Mexico	Homo sapiens		09-Oct-2014	
KU365367	ML	Mexico	Homo sapiens		07/nov/14	
KU365368	ML	Mexico	Homo sapiens		26/mar/14	
KJ451622	ML	Micronesia	Homo sapiens		Oct-2013	

KJ451623	ML	Micronesia	Homo sapiens		Oct-2013	
KJ689452	ML	Micronesia		Aedes aegypti mosquito pool	nov/13	
KJ689453	ML	Micronesia		Aedes hensilli mosquito pool	nov/13	
KY435457	ML	Montserrat	Homo sapiens	cell culture supernatant (vero)	30-Oct-2014	Passage in cell culture
KY435467	ML	Montserrat	Homo sapiens	cell culture supernatant (vero)	24/jul/14	Passage in cell culture
KF151174	ML	Myanmar	Homo sapiens	serum	13/jul/09	
KF151175	ML	Myanmar	Homo sapiens	serum	11-Dec-2009	
KF590564	ML	Myanmar	Homo sapiens	infected patient	2010	
KF590565	ML	Myanmar	Homo sapiens	infected patient	2010	
KF590566	ML	Myanmar	Homo sapiens	infected patient	2010	
KF590567	ML	Myanmar	Homo sapiens	infected patient	2010	
HE806461	ML	New-Caledonia	Homo sapiens	host serum	28-Feb-2011	
KT192707	ML	Nicaragua	Homo sapiens		31-Oct-2014	
KY703888	ML	Nicaragua	Homo sapiens		15-Aug-2015	passage history: not passaged;
KY703889	ML	Nicaragua	Homo sapiens		05-Aug-2015	passage history: not passaged;
KY703890	ML	Nicaragua	Homo sapiens		23-Sep-2015	passage history: not passaged;
KY703891	ML	Nicaragua	Homo sapiens		17-Sep-2015	passage history: not passaged;
KY703892	ML	Nicaragua	Homo sapiens		26/nov/15	passage history: not passaged;

KY703 893	ML	Nicarag ua	Homo sapiens		02-Sep-2015	passage history: unknown;
KY703 894	ML	Nicarag ua	Homo sapiens		09-Oct-2015	passage history: not passaged;
KY703 895	ML	Nicarag ua	Homo sapiens		26/no v/15	passage history: not passaged;
KY703 896	ML	Nicarag ua	Homo sapiens		18-Dec-2014	passage history: passaged;
KY703 897	ML	Nicarag ua	Homo sapiens		16/jan /15	passage history: not passaged;
KY703 898	ML	Nicarag ua	Homo sapiens		25-Aug-2015	passage history: not passaged;
KY703 899	ML	Nicarag ua	Homo sapiens		20-Oct-2015	passage history: not passaged;
KY703 900	ML	Nicarag ua	Homo sapiens		14-Dec-2015	passage history: not passaged;
KY703 901	ML	Nicarag ua	Homo sapiens		26/jan /15	passage history: not passaged;
KY703 902	ML	Nicarag ua	Homo sapiens		05-Sep-2015	passage history: unknown;
KY703 903	ML	Nicarag ua	Homo sapiens		05-Sep-2015	passage history: not passaged;
KY703 904	ML	Nicarag ua	Homo sapiens		04-Oct-2014	passage history: passaged;
KY703 905	ML	Nicarag ua	Homo sapiens		17-Sep-2015	passage history: not passaged;
KY703 906	ML	Nicarag ua	Homo sapiens		08-Sep-2015	passage history: not passaged;
KY703 907	ML	Nicarag ua	Homo sapiens		15/jul /15	passage history: not passaged;
KY703 908	ML	Nicarag ua	Homo sapiens		02-Dec-2014	passage history: passaged;

KY703 909	ML	Nicarag ua	Homo sapiens		28/jan /15	passage history: not passaged;
KY703 910	ML	Nicarag ua	Homo sapiens		18-Oct-2015	passage history: not passaged;
KY703 911	ML	Nicarag ua	Homo sapiens		17-Dec-2015	passage history: not passaged;
KY703 912	ML	Nicarag ua	Homo sapiens		14-Dec-2015	passage history: not passaged;
KY703 913	ML	Nicarag ua	Homo sapiens		13-Aug-2015	passage history: not passaged;
KY703 914	ML	Nicarag ua	Homo sapiens		15-Aug-2015	passage history: not passaged;
KY703 915	ML	Nicarag ua	Homo sapiens		03-Aug-2015	passage history: not passaged;
KY703 916	ML	Nicarag ua	Homo sapiens		04-Aug-2015	passage history: not passaged;
KY703 917	ML	Nicarag ua	Homo sapiens		08/jan /16	passage history: not passaged;
KY703 918	ML	Nicarag ua	Homo sapiens		26/jan /15	passage history: not passaged;
KY703 919	ML	Nicarag ua	Homo sapiens		29/jan /15	passage history: not passaged;
KY703 920	ML	Nicarag ua	Homo sapiens		16/no v/15	passage history: not passaged;
KY703 921	ML	Nicarag ua	Homo sapiens		04/no v/15	passage history: not passaged;
KY703 922	ML	Nicarag ua	Homo sapiens		06-Sep-2015	passage history: unknown;
KY703 923	ML	Nicarag ua	Homo sapiens		19-Sep-2015	passage history: not passaged;
KY703 924	ML	Nicarag ua	Homo sapiens		13-Aug-2015	passage history: not passaged;

KY703925	ML	Nicaragua	Homo sapiens		06-Sep-2015	passage history: unknown;
KY703926	ML	Nicaragua	Homo sapiens		20-Aug-2015	passage history: not passaged;
KY703927	ML	Nicaragua	Homo sapiens		15/jan/15	passage history: not passaged;
KY703928	ML	Nicaragua	Homo sapiens		30/jan/15	passage history: not passaged;
KY703929	ML	Nicaragua	Homo sapiens		02-Dec-2015	passage history: not passaged;
KY703930	ML	Nicaragua	Homo sapiens		24/nov/15	passage history: not passaged;
KY703931	ML	Nicaragua	Homo sapiens		19-Sep-2015	passage history: not passaged;
KY703932	ML	Nicaragua	Homo sapiens		19-Aug-2015	passage history: not passaged;
KY703933	ML	Nicaragua	Homo sapiens		09-Oct-2015	passage history: not passaged;
KY703934	ML	Nicaragua	Homo sapiens		27-Oct-2015	passage history: not passaged;
KY703935	ML	Nicaragua	Homo sapiens		02-Dec-2015	passage history: not passaged;
KY703936	ML	Nicaragua	Homo sapiens		07-Oct-2015	passage history: not passaged;
KY703937	ML	Nicaragua	Homo sapiens		15-Oct-2015	passage history: not passaged;
HM045786	ML	Nigeria	Homo sapiens		07/jul/64	
HM045807	ML	Nigeria	sentinel mouse	brain	13-Apr-1965	
KR559486	ML	Panama			nov/14	

AB860301	ML	Philippines	Homo sapiens	patient serum	2013	
HM045790	ML	Philippines	Homo sapiens		17/jul/85	
HM045800	ML	Philippines	Homo sapiens		1985	
KT308159	ML	Philippines	Homo sapiens	serum	2012	
KT308160	ML	Philippines	Homo sapiens	serum	2012	
KT308161	ML	Philippines	Homo sapiens	serum	2012	
KT308162	ML	Philippines	Homo sapiens	serum	2012	
KT308163	ML	Philippines	Homo sapiens	serum	2012	
KR264949	ML	Puerto-Rico	Homo sapiens		15/jul/14	
KR264950	ML	Puerto-Rico	Homo sapiens		16/jul/14	
KR264951	ML	Puerto-Rico	Homo sapiens		14-Aug-2014	
KR559470	ML	Puerto-Rico			nov/14	
KR559474	ML	Puerto-Rico			Sep-2014	
KR559483	ML	Puerto-Rico			Oct-2014	
KR559495	ML	Puerto-Rico			jul/14	
KF872195	ML	Russia	Homo sapiens	blood	24-Sep-2013	
KY435481	ML	Saint	Homo sapiens	cell culture supernatant (vero)	22-Apr-2014	Passage in cell culture
KY435482	ML	Saint	Homo sapiens	cell culture supernatant (vero)	11/mar/14	Passage in cell culture
KR559492	ML	Saint Lucia			Aug-2014	

KY435474	ML	Saint Lucia	Homo sapiens	cell culture supernatant (vero)	19-May-2014	Passage in cell culture
KX262991	ML	Saint Martin	Homo sapiens		2003	passage history: P2J3, Vero 2;
KY435473	ML	Saint Vincent	Homo sapiens	cell culture supernatant (vero)	22-May-2014	Passage in cell culture
KY435475	ML	Saint Vincent	Homo sapiens	cell culture supernatant (vero)	22-May-2014	Passage in cell culture
HM045785	ML	Senegal	Aedes aegypti		nov/66	
HM045798	ML	Senegal	Homo sapiens		nov/66	
HM045815	ML	Senegal	Aedes luteocephalus		Feb-1979	
HM045816	ML	Senegal	Homo sapiens		23/nov/66	
HM045817	ML	Senegal	Homo sapiens		nov/05	
HM045819	ML	Senegal	Aedes dalzieli		Feb-1993	
HM045821	ML/Bayes	Senegal	Chiroptera		20/mar/63	
KX262986	ML	Senegal	Aedes furcifer		1983	passage history: AP61#1, Vero 2;
KX262995	ML	Senegal	Aedes furcifer		10/jul/83	passage history: AP61#1, Vero1; genotype: West African
FJ445430	ML	Singapore	Homo sapiens	serum	jul/08	
FJ445431	ML	Singapore	Homo sapiens	serum	jul/08	
FJ445432	ML	Singapore	Homo sapiens	serum	jul/08	
FJ445433	ML	Singapore	Homo sapiens	serum	Aug-2008	
FJ445443	ML	Singapore	Homo sapiens	serum	Aug-2008	

FJ445445	ML	Singapore	Homo sapiens	serum	Aug-2008	
FJ445463	ML	Singapore	Homo sapiens	serum	jul/08	
FJ445484	ML	Singapore	Homo sapiens	serum	May-2008	
FJ445502	ML	Singapore	Homo sapiens	serum	Aug-2008	
FJ445510	ML	Singapore	Homo sapiens	serum	jan/08	
FJ445511	ML	Singapore	Homo sapiens	serum	jan/08	
FJ807896	ML	Singapore	Homo sapiens	infected patient	2006	
HM045792	ML/Bayes	South-Africa	Homo sapiens		Apr-1956	
HM045795	ML/Bayes	South-Africa	Homo sapiens		1976	
HM045805	ML/Bayes	South-Africa	Aedes furcifer		1976	
AB455493	ML	Sri-Lanka	Homo sapiens	infected patient	2006-12	passaged in Vero B4 cells
AB455494	ML	Sri-Lanka	Homo sapiens	infected patient	2006-12	passaged in Vero B4 cells
FJ445426	ML	Sri-Lanka	Homo sapiens	serum	Apr-2008	
FJ445427	ML	Sri-Lanka	Homo sapiens	serum	jul/07	
FJ445428	ML	Sri-Lanka	Homo sapiens	serum	May-2007	
FJ513628	ML	Sri-Lanka	Homo sapiens	serum	mar/08	
FJ513629	ML	Sri-Lanka	Homo sapiens	serum	mar/08	
FJ513632	ML	Sri-Lanka	Homo sapiens	serum	mar/08	
FJ513635	ML	Sri-Lanka	Homo sapiens	serum	mar/08	

FJ513637	ML	Sri-Lanka	Homo sapiens	serum	mar/08	
FJ513645	ML	Sri-Lanka	Homo sapiens	serum	Apr-2008	
FJ513654	ML	Sri-Lanka	Homo sapiens	serum	Apr-2008	
FJ513657	ML	Sri-Lanka	Homo sapiens	serum	Apr-2008	
FJ513673	ML	Sri-Lanka	Homo sapiens	serum	Apr-2008	
FJ513675	ML	Sri-Lanka	Homo sapiens	serum	Apr-2008	
FJ513679	ML	Sri-Lanka	Homo sapiens	serum	Apr-2008	
GU013528	ML	Sri-Lanka	Homo sapiens	serum	mar/08	
GU013529	ML	Sri-Lanka	Homo sapiens	serum	mar/08	
GU013530	ML	Sri-Lanka	Homo sapiens	serum	Apr-2008	
GU189061	ML	Sri-Lanka	Homo sapiens		2006	
HM045799	ML	Sri-Lanka	Homo sapiens		2007	
HM045801	ML	Sri-Lanka	Homo sapiens		2007	
KR559497	ML	St. Barts			jun/14	
KY435456	ML	Suriname	Homo sapiens	cell culture supernatant (vero)	17-Aug-2014	Passage in cell culture
KY435463	ML	Suriname	Homo sapiens	cell culture supernatant (vero)	02-Aug-2014	Passage in cell culture
HM045811	ML/Bayes	Tanzania	Homo sapiens		22-Feb-1953	
GQ905863	ML	Thailand	Homo sapiens		25-May-2009	
GU301779	ML	Thailand	Homo sapiens	serum	04-Sep-2009	

GU301780	ML	Thailand	Homo sapiens	serum	21-Oct-2008	
GU301781	ML	Thailand	Homo sapiens	culture	27/jul/09	Passage in cell culture
GU908223	ML	Thailand	Aedes albopictus	supernatant	14-Aug-2009	Passage in cell culture
HM045787	ML	Thailand	Homo sapiens		1995	
HM045789	ML	Thailand	Homo sapiens		1988	
HM045796	ML	Thailand	Homo sapiens		1995	
HM045808	ML	Thailand	Homo sapiens		1978	
HM045810	ML	Thailand	Homo sapiens		1958	
HM045814	ML	Thailand	Homo sapiens		1975	
KJ579184	ML	Thailand	Homo sapiens	serum sample from suspected patient	14-Oct-2013	
KJ579185	ML	Thailand	Homo sapiens	serum sample from suspected patient	14-Oct-2013	
KJ579186	ML	Thailand	Homo sapiens	serum sample from suspected patient	14-Oct-2013	
KJ579187	ML	Thailand	Homo sapiens	serum sample from suspected patient	14-Oct-2013	
KJ796847	ML	Thailand		serum	05/mar/09	
KJ796848	ML	Thailand		age 57 female serum	06/nov/08	
KJ796849	ML	Thailand		age 28 male serum	23-Dec-2008	
KJ796850	ML	Thailand		age 48 female serum	16-Sep-2009	

KJ7968 51	ML	Thailand		age 60 female serum	01-Dec-2008	
KJ7968 52	ML	Thailand		age 56 female serum	12-Feb-2009	
KP164 869	ML	Thailand			2009	
KX009 167	ML	Thailand	Homo sapiens	serum	2013	
KX009 168	ML	Thailand	Homo sapiens	serum	2013	
KX009 169	ML	Thailand	Homo sapiens	serum	2013	
KX009 170	ML	Thailand	Homo sapiens	serum	2013	
KX009 171	ML	Thailand	Homo sapiens	serum	2013	
KX262 987	ML	Thailand	Homo sapiens		1996	passage history: LLC-MK#2, Vero 1
KX262 988	ML	Thailand	Homo sapiens		1988	passage history: LLC-MK2#2,C636#1, Vero 1;
KR046 227	ML	Trinidad	Homo sapiens	serum	30-Aug-2014	
KR046 228	ML	Trinidad	Homo sapiens	serum	11-Sep-2014	
KR046 229	ML	Trinidad	Homo sapiens	serum	17-Sep-2014	
KR046 230	ML	Trinidad	Homo sapiens	serum	18-Sep-2014	
KR046 231	ML	Trinidad	Homo sapiens	serum	09/nov/14	
KR046 232	ML	Trinidad	Homo sapiens	serum	20-Sep-2014	
KR046 233	ML	Trinidad	Homo sapiens	serum	20-Sep-2014	

KR046234	ML	Trinidad	Homo sapiens	serum	12-Sep-2014	
KU365369	ML	Trinidad	Homo sapiens		2014	
KY435454	ML	Trinidad	Homo sapiens	cell culture supernatant (vero)	nov/11	Passage in cell culture
KY435465	ML	Trinidad	Homo sapiens	cell culture supernatant (vero)	17-Aug-2014	Passage in cell culture
KY435471	ML	Turks and Caicos	Homo sapiens	cell culture supernatant (vero)	11/jun/14	Passage in cell culture
KY435476	ML	Turks and Caicos	Homo sapiens	cell culture supernatant (vero)	05/jun/14	Passage in cell culture
HM045812	ML/Bayes	Uganda	Homo sapiens		1982	
HM045794	ML	USA	Homo sapiens		2006	
KJ941050	ML	USA	Homo sapiens	cell culture supernatant, Vero passage 4	2006	Passage in cell culture
KY575565	ML	USA	Homo sapiens		2014	passage history: V1;
KY575566	ML	USA	Homo sapiens		2014	passage history: V1;
KY575567	ML	USA	Homo sapiens		2006	passage history: P?V2
KY575568	ML	USA	Homo sapiens		2006	passage history: P?V2
KY575569	ML	USA	Homo sapiens		2014	passage history: V1;
KY575570	ML	USA	Homo sapiens		2008	passage history: V1
KY575571	ML	USA	Homo sapiens		2006	passage history: V2
KY575572	ML	USA	Homo sapiens		2014	passage history: V1;
KY575573	ML	USA	Homo sapiens		2014	passage history: V1;
KY575574	ML	USA	Homo sapiens		1995	passage history: P?SM1V3

KY680347	ML	USA	Homo sapiens		13-May-2014	passage history: VERO 0;
KY680348	ML	USA	Homo sapiens		05-Sep-2014	
KY680349	ML	USA	Homo sapiens		02/jul/14	passage history: VERO 0;
KY680350	ML	USA	Homo sapiens		10-Dec-2014	
KY680351	ML	USA	Homo sapiens		24/jun/14	passage history: VERO 0;
KY680352	ML	USA	Homo sapiens		15-May-2014	
KY680353	ML	USA	Homo sapiens		25-Aug-2014	
KY680354	ML	USA	Homo sapiens		08-May-2014	passage history: VERO 0;
KY680355	ML	USA	Homo sapiens		22/jul/14	
KY680356	ML	USA	Homo sapiens		21/jul/14	passage history: VERO 0;
KY680357	ML	USA	Homo sapiens		09-Aug-2014	passage history: VERO 0;
KY680358	ML	USA	Homo sapiens		08-Aug-2014	passage history: VERO 0;
KY680359	ML	USA	Homo sapiens		04/jul/14	passage history: VERO 0;
KY680360	ML	USA	Homo sapiens		05-Aug-2014	
KY680361	ML	USA	Homo sapiens		26-May-2014	
KY680362	ML	USA	Homo sapiens		26/jul/14	passage history: VERO 0;
KY680363	ML	USA	Homo sapiens		15/jun/14	passage history: VERO 0;

KY680 364	ML	USA	Homo sapiens		15-May-2014	
KY680 365	ML	USA	Homo sapiens		18-Sep-2014	
KY680 366	ML	USA	Homo sapiens		24/jul/15	
KY680 367	ML	USA	Homo sapiens		09/jun/14	passage history: VERO 0;
KY680 368	ML	USA	Homo sapiens		01-Dec-2014	
KY680 369	ML	USA	Homo sapiens		24-May-2014	passage history: VERO 0;
KY680 370	ML	USA	Homo sapiens		03-Oct-2014	
KY680 371	ML	USA	Homo sapiens		20-Aug-2014	passage history: VERO 0;
KY680 372	ML	USA	Homo sapiens		04/jun/14	passage history: VERO 0;
KY680 373	ML	USA	Homo sapiens		22-Oct-2014	
KY680 374	ML	USA	Homo sapiens		06-Dec-2014	
KY680 375	ML	USA	Homo sapiens		04/jun/14	passage history: VERO 0;
KY680 376	ML	USA	Homo sapiens		08-Oct-2014	
KY680 377	ML	USA	Homo sapiens		16-Aug-2014	
KY680 378	ML	USA	Homo sapiens		19-Oct-2014	
KY680 379	ML	USA	Homo sapiens		16-Sep-2014	

KY680380	ML	USA	Homo sapiens		13-Oct-2014	
KY680381	ML	USA	Homo sapiens		21-Oct-2014	
KY680382	ML	USA	Homo sapiens		04/jun/14	
KY680383	ML	USA	Homo sapiens		20-Aug-2014	
KY680384	ML	USA	Homo sapiens		25/jun/14	passage history: VERO 0;
KY680385	ML	USA	Homo sapiens		25/nov/14	
KY680386	ML	USA	Homo sapiens		24-Sep-2014	
KY680387	ML	USA	Homo sapiens		14-Oct-2014	
KY680388	ML	USA	Homo sapiens		02-Oct-2014	
KY680389	ML	USA	Homo sapiens		14/jul/15	
KY680390	ML	USA	Homo sapiens		07-May-2014	passage history: VERO 0;
KY680391	ML	USA	Homo sapiens		24-Oct-2014	
KY680392	ML	USA	Homo sapiens		19-Oct-2014	
KY680393	ML	USA	Homo sapiens		17-Sep-2014	
KY680394	ML	USA	Homo sapiens		05-Oct-2014	
KY680395	ML	USA	Homo sapiens		21/jul/14	passage history: VERO 0;

KY680 396	ML	USA	Homo sapiens		02-Sep-2014	
KY680 397	ML	USA	Homo sapiens		23-May-2014	passage history: VERO 0;
KY680 398	ML	USA	Homo sapiens		05-Aug-2014	
KY680 399	ML	USA	Homo sapiens		05-Sep-2014	
KY680 400	ML	USA	Homo sapiens		15/jul /14	passage history: VERO 0;
KY680 401	ML	USA	Homo sapiens		22-Sep-2014	
KY680 402	ML	USA	Homo sapiens		27/jul /14	passage history: VERO 0;
KY680 403	ML	USA	Homo sapiens		14/jun /14	passage history: VERO 0;
KY680 404	ML	USA	Homo sapiens		17-Sep-2014	
KY680 405	ML	USA	Homo sapiens		18/jun /14	passage history: VERO 0;
KY680 406	ML	USA	Homo sapiens		08-Aug-2014	passage history: VERO 0;
KY680 407	ML	USA	Homo sapiens		20/no v/14	
KY680 408	ML	USA	Homo sapiens		26/jun /14	passage history: VERO 0;
KY680 409	ML	USA	Homo sapiens		21-Oct-2014	
KY680 410	ML	USA	Homo sapiens		22-Aug-2014	passage history: VERO 0;
KY680 411	ML	USA	Homo sapiens		04-Sep-2014	

KY680412	ML	USA	Homo sapiens		28-Aug-2014	
KY680413	ML	USA	Homo sapiens		17-Sep-2014	
KY680414	ML	USA	Homo sapiens		13-Sep-2014	
MF001507	ML	USA	Homo sapiens		2015	passaged 1 time in C636
MF001508	ML	USA	Homo sapiens		2015	passaged 1 time in C636
MF001509	ML	USA	Homo sapiens		2015	passaged 1 time in C636
MF001510	ML	USA	Homo sapiens		2015	passaged 1 time in C636
MF001511	ML	USA	Homo sapiens		2015	passaged 1 time in C636
MF001512	ML	USA	Homo sapiens		2015	passaged 1 time in C636
MF001513	ML	USA	Homo sapiens		2015	passaged 1 time in C636
MF001514	ML	USA	Homo sapiens		2015	passaged 1 time in C636
MF001515	ML	USA	Homo sapiens		2015	passaged 1 time in C636
MF001516	ML	USA	Homo sapiens		2015	passaged 1 time in C636
MF001517	ML	USA	Homo sapiens		2015	passaged 1 time in C636
MF001518	ML	USA	Homo sapiens		2015	passaged 1 time in C636
MF001519	ML	USA	Homo sapiens		2015	passaged 1 time in C636
KU365374	ML	Venezuela	Homo sapiens		17-Dec-2014	
KR559480	ML	Virgin-Islands			Sep-2014	
KR559482	ML	Virgin-Islands			Aug-2014	

KR559 485	ML	Virgin-Islands			Oct-2014	
KR559 494	ML	Virgin-Islands			jul/14	
KC614 648	ML	Yemen	Aedes aegypti		25/jan /11	

ML: Maximum-likelihood; Bayes: Bayesian phylogeographic; USA: United States of America; CAR: Central African Republic. Empty spaces mean that these information are not public available.

Table S4 - Socio-Demographical data from Patients tested by RT-PCR

Year	2016	2017
Number of individual tested by RT-PCR (n)	827	999
Number of individual with detectable viral loads (n)	233	189
Detectable molecular diagnosis (%)		
CHIKV	72	37
DENV-4	12	17
ZIKV	16	47
Gender (%)		
Female	58	57
Age [median years-old (IQR)]	34 (23-45)	30 (21-45)
Rio de Janeiro's subregion (%, Figure S3 for reference)		
1.0	34	8
2.1	8	12
2.2	5	7
3.1	2	4
3.2	13	14
3.3	18	18
4.0	10	13
5.1	4	8
5.2	3	14
5.3	3	2

Table S5. Best fit spatial, molecular clock and demographic models for the ECSA Bayesian phylogeographic analysis.

Spatial	Clock	Demographic	PS	SS	Spatial			Clock			Demographic		
					Models	PS BF	SS BF	Models	PS BF	SS BF	Models	PS BF	SS BF
Sym	Strict	Skygrid	-23174,82	-23174,77	Sym/Assym	-3,46	14,47	Strict/UCLN	-6,14	-6,24	-	-	-
		Skyline	-23152,65	-23121,70		**9,93**	**40,96**		**8,33**	**39,45**	Skyl/Skyg	**22,17**	**53,07**
	UCLN	Skygrid	-23168,68	-23168,53		0,42	0,46	-	-	-	-	-	-
		Skyline	-23160,98	-23161,15		2,15	2,37		-	-	Skyl/Skyg	7,70	7,38
Assym	Strict	Skygrid	-23171,36	-23171,37	-	-	-	Strict/UCLN	-2,26	-2,39	-	-	-
		Skyline	-23162,58	-23162,67	-	-	-		0,55	0,85	Skyl/Skyg	8,78	8,71
	UCLN	Skygrid	-23169,10	-23168,99	-	-	-	-	-	-	-	-	-
		Skyline	-23163,13	-23163,52	-	-	-		-	-	Skyl/Skyg	5,97	5,47

Log marginal likelihood (ML) estimates for the phylogeographic models [symmetric (Sym) and asymmetric (Assym)], molecular-clock models [strict and uncorrelated log-normal (UCLN)], and non-parametric demographic models [Skygrid (Skyg) and Skyline (Skyl)] obtained using the path sampling (PS) and stepping-stone sampling (SS) methods. The Log Bayes factor (BF) is the difference of the Log ML between of alternative (H1) and null (H0) models (H1/H0). Log positive BFs values indicates that model H1 is more strongly supported by the data than model H0, while negative values indicate that BF is in favor of the model H0. Each model was systematically compared, and higher BF values were highlighted in bold while the best fit model was underlined.

Table S6 - Polymorphisms in the CHIKV genome

Mature peptide	Polymorphism Ty	Product	Nt Start	Nt Stop	CDS Type	CDS Positio	CDS Codon Numb	Protein position	Codon pos	Change	Polymorphism Ty	Codon Chang	Protein Effe	Amino Acid Chang	Variant Sequences
**Nonstructural protein nsP1 peptide**			1	1.605			1	1							
	SNP (transition)	nsP1	189		nonstructural	189	63	63	3	T -> C	SNP (transition)	GAT -> GAC	None		ECSA BR ROOT
	SNP (transition)	nsP1	267		nonstructural	267	89	89	3	T -> C	SNP (transition)	GAT -> GAC	None		ECSA AL NODE
	SNP (transition)	nsP1	309		nonstructural	309	103	103	3	C -> T	SNP (transition)	GCC -> GCT	None		ECSA BR ROOT
	SNP (transition)	nsP1	405		nonstructural	405	135	135	3	A -> G	SNP (transition)	TTA -> TTG	None		ECSA BR ROOT
	SNP (transition)	nsP1	603		nonstructural	603	201	201	3	T -> C	SNP (transition)	GAT -> GAC	None		ECSA BR ROOT
	SNP (transition)	nsP1	669		nonstructural	669	223	223	3	C -> T	SNP (transition)	GGC -> GGT	None		ECSA BR ROOT
	SNP (transition)	nsP1	708		nonstructural	708	236	236	3	C -> T	SNP (transition)	TGC -> TGT	None		ECSA BR ROOT
	SNP (transition)	nsP1	945		nonstructural	945	315	315	3	T -> C	SNP (transition)	TGT -> TGC	None		ECSA BR ROOT
	SNP (transition)	nsP1	1.389		nonstructural	1.389	463	463	3	T -> C	SNP (transition)	CCT -> CCC	None		ECSA BR ROOT
	SNP (transition)	nsP1	1.449		nonstructural	1.449	483	483	3	C -> T	SNP (transition)	TAC -> TAT	None		ECSA BR ROOT
**Non-structural protein nsP2 peptide; NTPase peptide**			1.606	3.999			536								
	SNP (transversion)	nsP2	1.623		nonstructural	1.623	541	6	3	G -> T	SNP (transversion)	CCG -> CCT	None		ECSA BR ROOT
	SNP (transition)	nsP2	1.785		nonstructural	1.785	595	60	3	T -> C	SNP (transition)	TAT -> TAC	None		ECSA BR ROOT
	SNP (transition)	nsP2	1.920		nonstructural	1.920	640	105	3	T -> C	SNP (transition)	CAT -> CAC	None		ECSA BR ROOT
	SNP (transition)	nsP2	2.169		nonstructural	2.169	723	188	3	G -> A	SNP (transition)	CCG -> CCA	None		ECSA BR ROOT
	SNP (transition)	nsP2	2.337		nonstructural	2.337	779	244	3	A -> G	SNP (transition)	AGA -> AGG	None		ECSA BR ROOT
	SNP (transition)	nsP2	2.436		nonstructural	2.436	812	277	3	T -> C	SNP (transition)	CTT -> CTC	None		ECSA BR ROOT
	**SNP (transversion)**	**nsP2**	**2.659**		**nonstructural**	**2.659**	**887**	**352**	**1**	**C -> G**	**SNP (transversion)**	**CCT -> GCT**	**Substitution**	**P -> A**	**ECSA AL NODE, ECSA RJ-SE NODE**
	SNP (transition)	nsP2	2.667		nonstructural	2.667	889	354	3	C -> T	SNP (transition)	GAC -> GAT	None		ECSA BR ROOT
	SNP (transition)	nsP2	2.778		nonstructural	2.778	926	391	3	C -> T	SNP (transition)	TAC -> TAT	None		ECSA BR ROOT
	SNP (transition)	nsP2	2.889		nonstructural	2.889	963	428	3	T -> C	SNP (transition)	GGT -> GGC	None		ECSA AL NODE
	SNP (transition)	nsP2	2.946		nonstructural	2.946	982	447	3	T -> C	SNP (transition)	ATT -> ATC	None		ECSA BR ROOT
	SNP (transition)	nsP2	3.033		nonstructural	3.033	1.011	476	3	C -> T	SNP (transition)	AAC -> AAT	None		ECSA BR ROOT
	SNP (transition)	nsP2	3.132		nonstructural	3.132	1.044	509	3	C -> T	SNP (transition)	GAC -> GAT	None		ECSA BR ROOT
	SNP (transition)	nsP2	3.193		nonstructural	3.193	1.065	530	1	C -> T	SNP (transition)	CTA -> TTA	None		ECSA BR ROOT
	SNP (transition)	nsP2	3.201		nonstructural	3.201	1.067	532	3	C -> T	SNP (transition)	AGC -> AGT	None		ECSA BR ROOT
	SNP (transition)	nsP2	3.219		nonstructural	3.219	1.073	538	3	G -> A	SNP (transition)	CCG -> CCA	None		ECSA BR ROOT
	SNP (transition)	nsP2	3.285		nonstructural	3.285	1.095	560	3	T -> C	SNP (transition)	TTT -> TTC	None		ECSA BR ROOT
	SNP (transition)	nsP2	3.369		nonstructural	3.369	1.123	588	3	T -> C	SNP (transition)	ACT -> ACC	None		ECSA BR ROOT
	SNP (transition)	nsP2	3.405		nonstructural	3.405	1.135	600	3	C -> T	SNP (transition)	AAC -> AAT	None		ECSA BR ROOT
	SNP (transition)	nsP2	3.420		nonstructural	3.420	1.140	605	3	C -> T	SNP (transition)	AAC -> AAT	None		ECSA BR ROOT
	SNP (transition)	nsP2	3.534		nonstructural	3.534	1.178	643	3	C -> T	SNP (transition)	AAC -> AAT	None		ECSA BR ROOT
	SNP (transition)	nsP2	3.539		nonstructural	3.539	1.180	645	2	C -> T	SNP (transition)	GCA -> GTA	Substitution	A -> V	ECSA BR ROOT
	SNP (transition)	nsP2	3.636		nonstructural	3.636	1.212	677	3	T -> C	SNP (transition)	GGT -> GGC	None		ECSA BR ROOT
	SNP (transition)	nsP2	3.825		nonstructural	3.825	1.275	740	3	A -> G	SNP (transition)	GTA -> GTG	None		ECSA BR ROOT
	SNP (transition)	nsP2	3.840		nonstructural	3.840	1.280	745	3	T -> C	SNP (transition)	TTT -> TTC	None		ECSA BR ROOT
	SNP (transition)	nsP2	3.951		nonstructural	3.951	1.317	782	3	C -> T	SNP (transition)	AAC -> AAT	None		ECSA BR ROOT
**Nonstructural protein nsP3 peptide**			4.000	5.589			1334								
	SNP (transition)	nsP3	4.059		nonstructural	4.059	1.353	20	3	C -> T	SNP (transition)	GTC -> GTT	None		ECSA BR ROOT
	SNP (transition)	nsP3	4.173		nonstructural	4.173	1.391	58	3	T -> C	SNP (transition)	GTT -> GTC	None		ECSA BR ROOT
	SNP (transition)	nsP3	4.212		nonstructural	4.212	1.404	71	3	A -> G	SNP (transition)	CCA -> CCG	None		ECSA BR ROOT
	SNP (transition)	nsP3	4.215		nonstructural	4.215	1.405	72	3	C -> T	SNP (transition)	AAC -> AAT	None		ECSA RJ-SE NODE
	SNP (transition)	nsP3	4.229		nonstructural	4.229	1.410	77	2	C -> T	SNP (transition)	TCG -> TTG	Substitution	S -> L	ECSA BR ROOT
	SNP (transition)	nsP3	4.233		nonstructural	4.233	1.411	78	3	G -> A	SNP (transition)	GAG -> GAA	None		ECSA BR ROOT
	SNP (transition)	nsP3	4.356		nonstructural	4.356	1.452	119	3	C -> T	SNP (transition)	GAC -> GAT	None		ECSA BR ROOT
	SNP (transition)	nsP3	4.386		nonstructural	4.386	1.462	129	3	T -> C	SNP (transition)	TTT -> TTC	None		ECSA BR ROOT
	SNP (transition)	nsP3	4.428		nonstructural	4.428	1.476	143	3	C -> T	SNP (transition)	TGC -> TGT	None		ECSA BR ROOT
	SNP (transition)	nsP3	4.489		nonstructural	4.489	1.497	164	1	C -> T	SNP (transition)	CTG -> TTG	None		ECSA BR ROOT
	SNP (transition)	nsP3	4.599		nonstructural	4.599	1.533	200	3	T -> C	SNP (transition)	TAT -> TAC	None		ECSA AL NODE
	SNP (transition)	nsP3	4.611		nonstructural	4.611	1.537	204	3	C -> T	SNP (transition)	ACC -> ACT	None		ECSA BR ROOT
	SNP (transition)	nsP3	4.635		nonstructural	4.635	1.545	212	3	T -> C	SNP (transition)	GAT -> GAC	None		ECSA BR ROOT
	SNP (transition)	nsP3	4.641		nonstructural	4.641	1.547	214	3	G -> A	SNP (transition)	GCG -> GCA	None		ECSA BR ROOT
	SNP (transition)	nsP3	4.761		nonstructural	4.761	1.587	254	3	A -> G	SNP (transition)	TCA -> TCG	None		ECSA BR ROOT
	SNP (transition)	nsP3	4.767		nonstructural	4.767	1.589	256	3	C -> T	SNP (transition)	CCC -> CCT	None		ECSA BR ROOT
	SNP (transversion)	nsP3	4.770		nonstructural	4.770	1.590	257	3	C -> A	SNP (transversion)	CCC -> CCA	None		ECSA BR ROOT
	SNP (transition)	nsP3	4.809		nonstructural	4.809	1.603	270	3	A -> G	SNP (transition)	CCA -> CCG	None		ECSA BR ROOT
	SNP (transition)	nsP3	4.848		nonstructural	4.848	1.616	283	3	C -> T	SNP (transition)	AGC -> AGT	None		ECSA BR ROOT
	SNP (transition)	nsP3	4.863		nonstructural	4.863	1.621	288	3	T -> C	SNP (transition)	TCT -> TCC	None		ECSA BR ROOT
	**SNP (transition)**	**nsP3**	**4.950**		**nonstructural**	**4.950**	**1.650**	**317**	**3**	**G -> A**	**SNP (transition)**	**TCG -> TCA**	**None**		**ECSA BR ROOT, ECSA RJ-SE NODE**

	SNP (transversion)	nsP3	4.987		nonstructural	4.987	1.663	330	1	T -> G	SNP (transversion)	TCT -> GCT	Substitution	S -> A	ECSA BR ROOT
	SNP (transition)	nsP3	5.061		nonstructural	5.061	1.687	354	3	A -> G	SNP (transition)	CTA -> CTG	None		ECSA AL NODE
	SNP (transition)	nsP3	5.109		nonstructural	5.109	1.703	370	3	C -> T	SNP (transition)	GCC -> GCT	None		ECSA BR ROOT
	SNP (transition)	nsP3	5.119		nonstructural	5.119	1.707	374	1	G -> A	SNP (transition)	GGG -> AGG	Substitution	G -> R	ECSA BR ROOT
	SNP (transition)	nsP3	5.148		nonstructural	5.148	1.716	383	3	T -> C	SNP (transition)	ACT -> ACC	None		ECSA BR ROOT
	SNP (transition)	nsP3	5.345		nonstructural	5.345	1.782	449	2	C -> T	SNP (transition)	ACG -> ATG	Substitution	T -> M	ECSA BR ROOT
**Nonstructural protein nsP4 peptide**			5.590	7.422			1864								
	SNP (transition)	nsP4	5.707		nonstructural	5.707	1.903	40	1	C -> T	SNP (transition)	CTG -> TTG	None		ECSA BR ROOT
	SNP (transition)	nsP4	5.717		nonstructural	5.717	1.906	43	2	C -> T	SNP (transition)	GCA -> GTA	Substitution	A -> V	ECSA BR ROOT
	SNP (transition)	nsP4	5.866		nonstructural	5.866	1.956	93	1	C -> T	SNP (transition)	CCA -> TCA	Substitution	P -> S	ECSA BR ROOT
	SNP (transition)	nsP4	5.920		nonstructural	5.920	1.974	111	1	A -> G	SNP (transition)	ATC -> GTC	Substitution	I -> V	ECSA RJ NODE
	SNP (transition)	nsP4	6.034		nonstructural	6.034	2.012	149	1	C -> T	SNP (transition)	CTA -> TTA	None		ECSA BR ROOT
	SNP (transition)	nsP4	6.126		nonstructural	6.126	2.042	179	3	C -> T	SNP (transition)	CAC -> CAT	None		ECSA BR ROOT
	SNP (transition)	nsP4	6.273		nonstructural	6.273	2.091	228	3	C -> T	SNP (transition)	TTC -> TTT	None		ECSA BR ROOT
	SNP (transition)	nsP4	6.396		nonstructural	6.396	2.132	269	3	C -> T	SNP (transition)	TTC -> TTT	None		ECSA BR ROOT
	SNP (transition)	nsP4	6.408		nonstructural	6.408	2.136	273	3	T -> C	SNP (transition)	CAT -> CAC	None		ECSA BR ROOT
	SNP (transition)	nsP4	6.630		nonstructural	6.630	2.210	347	3	C -> T	SNP (transition)	GAC -> GAT	None		ECSA BR ROOT
	SNP (transition)	nsP4	6.717		nonstructural	6.717	2.239	376	3	T -> C	SNP (transition)	GAT -> GAC	None		ECSA BR ROOT
	SNP (transition)	nsP4	6.750		nonstructural	6.750	2.250	387	3	T -> C	SNP (transition)	GCT -> GCC	None		ECSA BR ROOT
	SNP (transition)	nsP4	6.757		nonstructural	6.757	2.253	390	1	C -> T	SNP (transition)	CTG -> TTG	None		ECSA BR ROOT
	SNP (transition)	nsP4	6.813		nonstructural	6.813	2.271	408	3	C -> T	SNP (transition)	TTC -> TTT	None		ECSA BR ROOT
	SNP (transversion)	nsP4	6.936		nonstructural	6.936	2.312	449	3	A -> C	SNP (transversion)	CGA -> CGC	None		ECSA BR ROOT
	SNP (transversion)	nsP4	7.031		nonstructural	7.031	2.344	481	2	C -> A	SNP (transversion)	GCC -> GAC	Substitution	A -> D	ECSA RJ-SE NODE
	SNP (transition)	C protein	7.146		nonstructural	7.146	2.382	519	3	T -> C	SNP (transition)	GCT -> GCC	None		ECSA BR ROOT
**C protein peptide**			7.491	8.273		1	1	1							
	SNP (transversion)	C protein	7.498		structural	8	3	3	2	T -> A	SNP (transversion)	TTC -> TAC	Substitution	F -> Y	ECSA BR ROOT
	SNP (transition)	C protein	7.559		structural	69	23	23	3	T -> C	SNP (transition)	CCT -> CCC	None		ECSA BR ROOT
	SNP (transversion)	C protein	7.571		structural	81	27	27	3	C -> A	SNP (transversion)	GTC -> GTA	None		ECSA BR ROOT
	SNP (transition)	C protein	7.604		structural	114	38	38	3	T -> C	SNP (transition)	GCT -> GCC	None		ECSA BR ROOT
	SNP (transition)	C protein	7.711		structural	221	74	74	2	A -> G	SNP (transition)	CAA -> CGA	Substitution	Q -> R	ECSA BR ROOT
	SNP (transition)	C protein	7.835		structural	345	115	115	3	C -> T	SNP (transition)	TTC -> TTT	None		ECSA BR ROOT
	SNP (transition)	C protein	7.845		structural	355	119	119	1	C -> T	SNP (transition)	CAT -> TAT	Substitution	H -> Y	ECSA BR ROOT
	SNP (transition)	C protein	8.039		structural	549	183	183	3	G -> A	SNP (transition)	CCG -> CCA	None		ECSA BR ROOT
**E3 protein peptide**			8.274	8.465			262								
	SNP (transition)	E3	8.301		structural	811	271	10	1	T -> C	SNP (transition)	TTG -> CTG	None		ECSA BR ROOT
	SNP (transition)	E3	8.309		structural	819	273	12	3	C -> T	SNP (transition)	AAC -> AAT	None		ECSA BR ROOT
	SNP (transversion)	E3	8.342		structural	852	284	23	3	A -> T	SNP (transversion)	ACA -> ACT	None		ECSA BR ROOT
	SNP (transition)	E3	8.354		structural	864	288	27	3	C -> T	SNP (transition)	TAC -> TAT	None		ECSA BR ROOT
	SNP (transition)	E3	8.359		structural	869	290	29	2	A -> G	SNP (transition)	AAG -> AGG	Substitution	K -> R	ECSA BR ROOT
	SNP (transition)	E3	8.456		structural	966	322	61	3	C -> T	SNP (transition)	CGC -> CGT	None		ECSA BR ROOT
**E2 protein peptide**			8.466	9.734			326								
	SNP (transition)	E2	8.507		structural	1.017	339	14	3	A -> G	SNP (transition)	CCA -> CCG	None		ECSA BR ROOT
	SNP (transition)	E2	8.565		structural	1.075	359	34	1	C -> T	SNP (transition)	CTA -> TTA	None		ECSA BR ROOT
	SNP (transition)	E2	8.686		structural	1.196	399	74	2	T -> C	SNP (transition)	ATG -> ACG	Substitution	M -> T	ECSA BR ROOT
	SNP (transition)	E2	8.693		structural	1.203	401	76	3	A -> G	SNP (transition)	GCA -> GCG	None		ECSA BR ROOT
	SNP (transition)	E2	8.772		structural	1.282	428	103	1	G -> A	SNP (transition)	GCC -> ACC	Substitution	A -> T	ECSA BR ROOT
	SNP (transition)	E2	8.858		structural	1.368	456	131	3	C -> T	SNP (transition)	CAC -> CAT	None		ECSA AL NODE
	SNP (transition)	E2	8.882		structural	1.392	464	139	3	A -> G	SNP (transition)	GAA -> GAG	None		ECSA BR ROOT
	SNP (transition)	E2	8.918		structural	1.428	476	151	3	A -> G	SNP (transition)	CTA -> CTG	None		ECSA BR ROOT
	SNP (transition)	E2	9.041		structural	1.551	517	192	3	T -> C	SNP (transition)	GTT -> GTC	None		ECSA AL NODE
	SNP (transition)	E2	9.128		structural	1.638	546	221	3	G -> A	SNP (transition)	AAG -> AAA	None		ECSA BR ROOT
	SNP (transition)	E2	9.176		structural	1.686	562	237	3	T -> C	SNP (transition)	TAT -> TAC	None		ECSA BR ROOT
	SNP (transition)	E2	9.230		structural	1.740	580	255	3	T -> C	SNP (transition)	ATT -> ATC	None		ECSA BR ROOT
	SNP (transition)	E2	9.332		structural	1.842	614	289	3	T -> C	SNP (transition)	CCT -> CCC	None		ECSA BR ROOT
	SNP (transition)	E2	9.362		structural	1.872	624	299	3	T -> C	SNP (transition)	AAT -> AAC	None		ECSA BR ROOT
	SNP (transition)	E2	9.383		structural	1.893	631	306	3	T -> C	SNP (transition)	TAT -> TAC	None		ECSA BR ROOT
	SNP (transition)	E2	9.458		structural	1.968	656	331	3	C -> T	SNP (transition)	GGC -> GGT	None		ECSA BR ROOT
**6K protein peptide**			9.735	9.917			749								
	SNP (transition)	6k	9.770		structural	2.280	760	12	3	C -> T	SNP (transition)	AAC -> AAT	None		ECSA BR ROOT
	SNP (transition)	6k	9.773		structural	2.283	761	13	3	G -> A	SNP (transition)	GAG -> GAA	None		ECSA RJ NODE
	SNP (transition)	6k	9.860		structural	2.370	790	42	3	T -> C	SNP (transition)	TGT -> TGC	None		ECSA BR ROOT
**E1 protein peptide**			9.918	11.234			810								
	SNP (transition)	E1	10.079		structural	2.589	863	54	3	C -> T	SNP (transition)	GTC -> GTT	None		ECSA BR ROOT

	SNP (transversion)	E1	10.190		structural	2.700	900	91	3	C -> A	SNP (transversion)	GGC -> GGA	None		ECSA BR ROOT
	SNP (transition)	E1	10.238		structural	2.748	916	107	3	T -> C	SNP (transition)	CAT -> CAC	None		ECSA BR ROOT
	SNP (transversion)	E1	10.361		structural	2.871	957	148	3	A -> T	SNP (transversion)	GCA -> GCT	None		ECSA BR ROOT
	SNP (transversion)	E1	10.549		structural	3.059	1.020	211	2	A -> C	SNP (transversion)	AAA -> ACA	Substitution	K -> T	ECSA RJ-SE NODE
	SNP (transition)	E1	10.727		structural	3.237	1.079	270	3	C -> T	SNP (transition)	AAC -> AAT	None		ECSA BR ROOT
	SNP (transition)	E1	10.780		structural	3.290	1.097	288	2	C -> T	SNP (transition)	ACT -> ATT	Substitution	T -> I	ECSA BR ROOT
	SNP (transversion)	E1	10.931		structural	3.441	1.147	338	3	T -> A	SNP (transversion)	ACT -> ACA	None		ECSA BR ROOT
	SNP (transition)	E1	10.997		structural	3.507	1.169	360	3	A -> G	SNP (transition)	TTA -> TTG	None		ECSA BR ROOT
	SNP (transition)	E1	11.047		structural	3.557	1.186	377	2	C -> T	SNP (transition)	GCA -> GTA	Substitution	A -> V	ECSA BR ROOT
	SNP (transversion)	E1	11.136		structural	3.646	1.216	407	1	A -> C	SNP (transversion)	ATG -> CTG	Substitution	M -> L	ECSA BR ROOT
	SNP (transition)	E1	11.165		structural	3.675	1.225	416	3	T -> C	SNP (transition)	GGT -> GGC	None		ECSA BR ROOT
	SNP (transition)	E1	11.225		structural	3.735	1.245	436	3	T -> C	SNP (transition)	TTT -> TTC	None		ECSA BR ROOT
